# Assessment of temperature dynamics in pulsed field ablation with a variable-loop circular catheter: a comparative analysis of waveform configurations and irrigation rates in specimens of bovine ventricular myocardium

**DOI:** 10.1093/europace/euaf278

**Published:** 2025-10-30

**Authors:** Elio Zito, Moussa Mansour, Vivek Y Reddy, Amin Al-Ahmad, Vincenzo Mirco La Fazia, Carola Gianni, J David Burkhardt, Sanghamitra Mohanty, Thanassis Papaioannou, Tushar Sharma, Luigi Di Biase, Andrea Natale

**Affiliations:** Texas Cardiac Arrhythmia Institute, St David’s Medical Center, 3000 N Interstate 35, Suite 700, Austin, TX 78705, USA; Department of Biomedical, Surgical and Dental Sciences, University of Milan, Via Festa del Perdono 7, Milan, MI 20122, Italy; Heart Center, Massachusetts General Hospital, Boston, MA, USA; Department of Cardiology, Icahn School of Medicine at Mount Sinai, One Gustave Levy Place, Box 1030, New York, NY, USA; Texas Cardiac Arrhythmia Institute, St David’s Medical Center, 3000 N Interstate 35, Suite 700, Austin, TX 78705, USA; Texas Cardiac Arrhythmia Institute, St David’s Medical Center, 3000 N Interstate 35, Suite 700, Austin, TX 78705, USA; Department of Biomedicine and Prevention, Division of Cardiology, University of Tor Vergata, Rome, Italy; Texas Cardiac Arrhythmia Institute, St David’s Medical Center, 3000 N Interstate 35, Suite 700, Austin, TX 78705, USA; Texas Cardiac Arrhythmia Institute, St David’s Medical Center, 3000 N Interstate 35, Suite 700, Austin, TX 78705, USA; Texas Cardiac Arrhythmia Institute, St David’s Medical Center, 3000 N Interstate 35, Suite 700, Austin, TX 78705, USA; Biosense Webster, Irvine, CA, USA; Biosense Webster, Irvine, CA, USA; Texas Cardiac Arrhythmia Institute, St David’s Medical Center, 3000 N Interstate 35, Suite 700, Austin, TX 78705, USA; Department of Electrophysiology, Albert Einstein College of Medicine, New York, NY, USA; Texas Cardiac Arrhythmia Institute, St David’s Medical Center, 3000 N Interstate 35, Suite 700, Austin, TX 78705, USA; Department of Biomedicine and Prevention, Division of Cardiology, University of Tor Vergata, Rome, Italy; Interventional Electrophysiology, Scripps Clinic, San Diego, CA, USA; Metro Health Medical Center, Case Western Reserve University School of Medicine, Cleveland, OH, USA

**Keywords:** Atrial fibrillation, Catheter ablation, Pulse field ablation, Heating, VLCC

## Abstract

**Aims:**

Pulsed Field ablation (PFA) is a non-thermal ablation modality with functional myocardial sparing. Recent evidence suggests that clinically used PFA systems may produce non-negligible thermal effects, particularly at the tissue-catheter interface, potentially increasing the risk of thrombo-embolic complications. This study sought to characterize the temperature dynamics of two PFA pulse waveforms, delivered using a variable-loop circular catheter, under different irrigation conditions.

**Methods and results:**

In total, 132 ablations were performed on 31 bovine myocardial tissue specimens, using Sequence_1 and Sequence_2 at two different irrigation rates—4 and 30 mL/min. Maximum temperatures and their rise from baseline were measured at the tissue surface, and at 3 and 7 mm depths, across different ablation conditions. Sequence_1 at 4 mL/min produced the highest surface temperature (56.4°C [54.9–58.4]) and temperature rise (Δ*T*: 19.4°C [17.9–21.4]). Sequence_2 at 30 mL/min showed the most favourable thermal profile, with significantly lower surface temperatures (40.8°C [37.9–43.0], Δ*T*: 3.8°C [0.9–6.0], *P* < 0.0001). At 3 mm depth, temperature increases were reduced for all settings, but remained highest with Sequence_1 at 4 mL/min (42.5°C, Δ*T*: 5.5°C). At 7 mm depth, temperatures remained close to baseline. Both waveform and irrigation optimization independently and synergistically reduced tissue heating, with the Sequence_2 at 30 mL/min achieving the lowest thermal load at all depths.

**Conclusion:**

This study confirms that PFA can induce relevant thermal effects, especially at the tissue interface. However, waveform optimization and active cooling significantly mitigate these effects. Such strategies to minimize thermal effects should be implemented in clinical practice to enhance procedural safety.

## Graphical Abstract

**Graphical Abstract:**
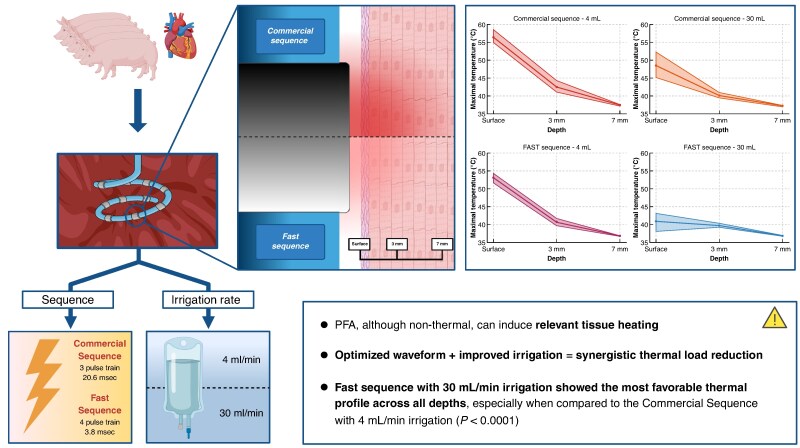

What’s new?

- To the best of our knowledge, this is the first study to systematically quantify surface and subsurface temperature dynamics during PFA using a commercially available variable-loop circular PFA catheter, under clinically relevant ablation settings.
- The commercial pulse waveform (Sequence 1) produced significant surface heating, with peak temperatures exceeding 60°C under low irrigation, while the experimental waveform (fast Sequence 2) combined with high irrigation (30 mL/min) significantly reduced surface and deep tissue temperatures.
- Optimization of both pulse waveform characteristics and irrigation flow rate resulted in a synergistic reduction in thermal load across all tissue depths.
- These findings highlight the need to reassess the thermal safety profile of current PFA systems and support the implementation of waveform and irrigation modifications to mitigate heat-related complications.

## Introduction

Catheter ablation for atrial fibrillation (AF) has evolved substantially over the past 25 years, with the advent of successive innovations in energy sources and catheter technologies transforming and optimizing daily clinical practice.^1,2^ Pulse field ablation (PFA) is a novel energy source based on the principle of irreversible electroporation.^3^ Preclinical and clinical studies have demonstrated that PFA avoids significant collateral damage to many surrounding anatomical structures, such as nerves and the oesophagus.^4,5^ While its mechanism is largely non-thermal, PFA can produce a variable amount of collateral heat. When this exceeds a critical threshold, energy delivery can charge the medium (saline solution or blood), potentially resulting in temperature rises, which may in turn lead to microbubble formation, catheter overheating, and eventually charring.^6^ These last two events have been largely described during early stages of radiofrequency (RF) ablation and have been considered surrogates of the intrinsic embolic predisposition of a particular device. In fact, the by-products responsible for catheter charring—mainly blood clots and burned tissue—and *large* microbubbles, can act as sources for thrombo-embolic events.^7^

Heat generation during PFA has been described to occur especially in catheter regions with higher electrical field strengths in several *in vitro* and *in vivo* experimental studies.^6,7^ Multiple factors can influence electrical field-related heat dissipation, including catheter design, electrode geometry (dimension and spacing), pulse waveforms’ characteristics (i.e. monophasic/biphasic, duty cycle), number of pulses, and the energy level used.^6,8–11^ Therefore, the non-thermal nature of PFA energy should not be considered a qualitative feature, but rather a quantitative one. Two recently published preclinical studies suggested that some of the current commercially available PFA systems may have the potential to produce detectable heating, particularly with repeated applications of pulsed field energy.^12,13^ While temperature elevations beyond the oesophageal safety threshold appear rare,^13^ these studies further raise questions about the true extent of overheating by PFA catheters and the potential local consequences. Experimental data on temperature profiles during PFA remain limited, and the thermal characteristics of each PFA system should be individually evaluated. Therefore, the purpose of this study was to characterize tissue temperature profiles generated using two different pulse waveforms—one commercial and one experimental—available with the standard commercially available variable-loop biphasic circular PFA catheter under two different irrigation conditions.

## Methods

### Experimental design

The purpose of this study was to assess the change in tissue temperature during PFA with the variable-loop circular PFA catheter (VLCC) under the commercial ablation Sequence 1 and the fast ablation Sequence 2 for two irrigation rates of 4 and 30 mL/min, in a controlled *in vitro* model.

### Pulsed field ablation system and ablation catheter

The ablation system is composed by a circular catheter (Varipulse; JNJ MedTech Inc., Irvine, CA, USA) and a proprietary PFA generator (Trupulse; JNJ MedTech Inc.), both compatible with the Carto 3 electro-anatomical mapping system (JNJ MedTech Inc.) The 7.5F circular catheter includes 10 electrodes with individual irrigation pores and has an adjustable diameter between 25 and 35 mm. Pulse field ablation is applied in a bipolar configuration between skipped electrodes (i.e. electrode 1 to electrode 3) and between each of the adjacent electrodes (i.e. electrodes 1–2 and 2–3). Each PFA application includes trains of microsecond-long biphasic pulses between all bipolar configurations.^14^

### Waveform characteristics

Ablations were conducted with the commercial Sequence 1 and the experimental Sequence 2 at both 4 and 30 mL/min irrigation rates with 30 g applied force. Commercial Sequence 1 is characterized by 3 pulse trains with a total estimated duration of single ablation of 20.6 s, whereas the optimized fast Sequence 2 delivers 4 pulse trains with a reduced duration of 3.8 s. Each ablation consisted of one application. *Table 1* shows the ablation settings of the two different waveforms used.

**Table 1:** Ablation parameters

Ablation sequence	Setting	Duration of single ablation (sec)	Irrigation during ablation (mL/min)
Sequence 1 (commercial)	3 pulse train	20.6	4
Sequence 1 (commercial)			30
Sequence 2 (fast)	4 pulse train	3.8	4
Sequence 2 (fast)			30

### Tissue and saline bath preparation

Fresh bovine hearts were obtained from a USDA-compliant slaughterhouse through a certified supplier (Sierra for Medical Science). Animals were disease-free, aged 20–30 months, and had an average weight of 1200–1500 lbs. As only post-mortem tissue was used, no IACUC approval was required. Upon receipt, the hearts were refrigerated. Free ventricular wall and septal tissues were excised from bovine hearts and cut into sections ranging from 111.43 mm × 111.43 mm to 63.5 mm × 76.2 mm in size. A total of 31 tissue preparations were used. The tissue was placed in a ∼37°C temperature-controlled saline bath and was allowed to thermally equilibrate with it. Temperature control was achieved by saline circulation at constant flow, ensuring heat exchange with an external heat bath. No directed surface flow was applied onto the catheter tip or toward the ablated area, thereby providing no direct cooling to the tissue surface.

### Catheter and temperature probe positioning

**Figure 1:**
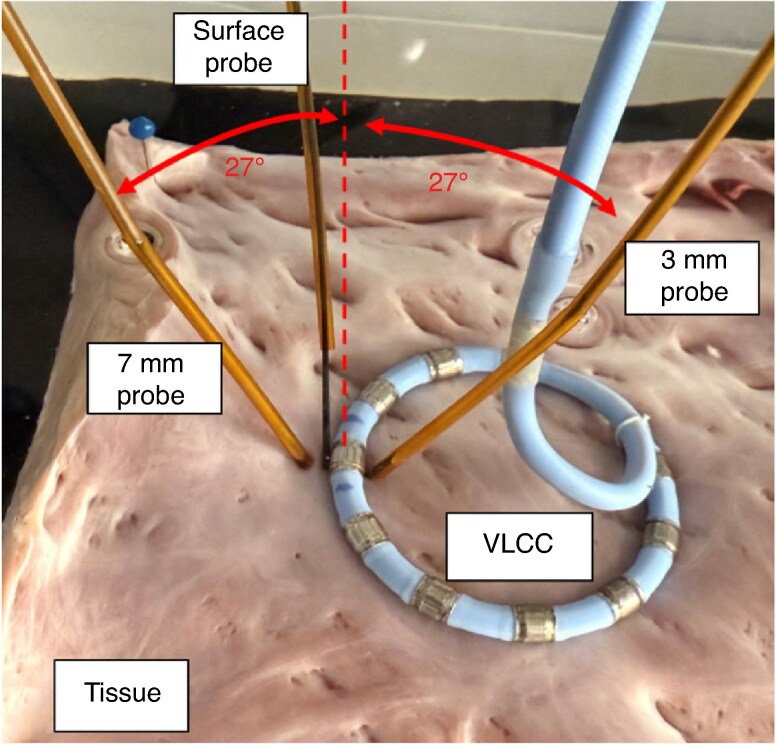
Placement of the temperature probes with respect to a single electrode of the VLCC. VLCC, variable-loop circular catheter.

Ablations were conducted with the VLCC’s 10-electrode loop placed in contact to the endocardial tissue. The two depth probes were inserted into the tissue at an angle of 27° with respect to the main axis of the catheter. The surface probe sensing tip was placed at the interface between and in contact with the electrode and the underlying tissue. *Figure 1* shows the general placement of the three temperature probes. Two pilot holes were initially made into the tissue to facilitate insertion of the two depth probes. The holes were made with a needle oriented at 27° relative to the perpendicular of the tissue surface. Following removal of the needle, each probe was inserted into the corresponding pilot hole.

**Figure 2:**
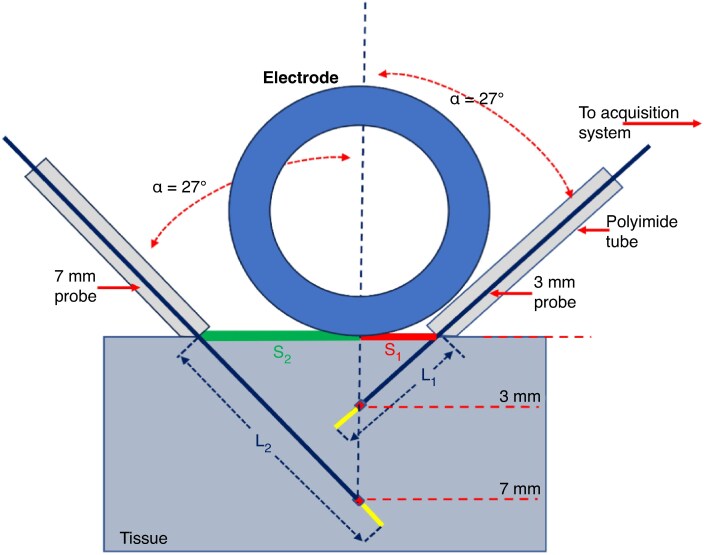
Cross-sectional schematic showing the depth probe placement for the VLCC catheter. VLCC, variable-loop circular catheter.

To achieve consistency of temperature probe placement, the following was done: (1) a polyimide tube of 1.2 mm in outer diameter was securely fitted over the distal section of each temperature probe, leaving a predetermined length of the temperature probe exposed (*L*_1_ for the 3 mm and *L*_2_ for the 7 mm probe, respectively); (2) each depth temperature probe was inserted into the tissue at an angle (*α*) of ∼ 27° with respect to the main axis of the catheter (*Figure 2*). This angle and the required depth of 3 mm or 7 mm determined the length *L*_1_ or *L*_2_, which was calculated by the following equations (1 and 2):


(1) $$L_{1}=0.7\;\mathrm{mm}+3\;\mathrm{mm}/\cos\;(27^{\circ })=4.1\;\mathrm{mm}$$

(2) $$L_{2}=0.5\;\mathrm{mm}+7\;\mathrm{mm}/\cos\;(27^{\circ })=8.4\;\mathrm{mm}$$

where 0.7 and 0.5 mm represent the distance (yellow section in *Figure 2*) of the sensing area from the end of the 3 and 7 mm depth probe, respectively. The distances *S*_1_ and *S*_2_, between the centre of the electrode tip and the corresponding probes entry site, determined the proper alignment of the sensing section of the probe with the centre of the electrode and were calculated with the following equations (3 and 4):


(3) $$S_{1}=3\;\mathrm{mm}\times \tan\;(27^{\circ })=1.5\;\mathrm{mm}$$

(4) $$S_{2}=7\;\mathrm{mm}\times \tan\;(27^{\circ })=3.6\;\mathrm{mm}$$

The distal section of each catheter was attached to a balanced arm and set to provide 30 g of constant force. The entrance ports of the two probes were 5.1 mm (= *S*_1_ + *S*_2_) apart. Following insertion of each probe at 27° inclination, they were advanced into the tissue, and the appropriate depth (*L*_1_, *L*_2_) was established. This was assured by the oversized polyimide tubing, which acted as a stop. The catheter was then placed so that its loop was in contact with the tissue surface, while the investigated electrode was placed along the line between the two probes and tangent to the polyimide surface of the 3 mm probe. Accounting for the electrode size and the polyimide outer diameter, this arrangement ensured proper depth and centration of each probe.

### Characterization of the temperature acquisition system

**Figure 3:**
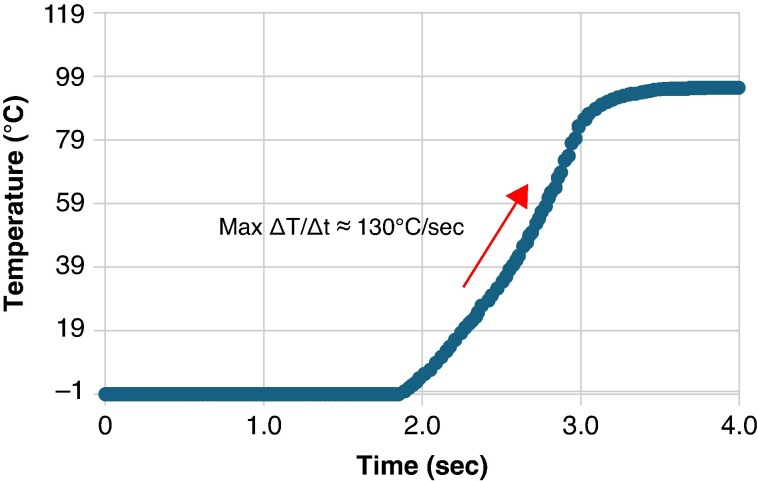
Typical time–response curve of the temperature acquisition system as the probe experiences an abrupt temperature step from iced water (∼0°C) to a bath of boiling water (∼100°C).

The temperature acquisition system used 0.5 mm fiberoptic-based fluoroptic temperature probes and an acquisition system capable of a 50 Hz sampling rate (sampling interval: 20 ms). Temperature probe verification was conducted a day before testing. The time response of the system (probe and acquisition kit) was characterized by exposing the probe to a step change in temperature from iced water temperature (∼0°C) into a bath of boiling water (100°C). The temporal response was estimated by calculation of the maximum slope of the resulting temperature curve (*Figure 3*) and was found to be 130°C/s.

### Determination of initiation of ablation

**Figure 4:**
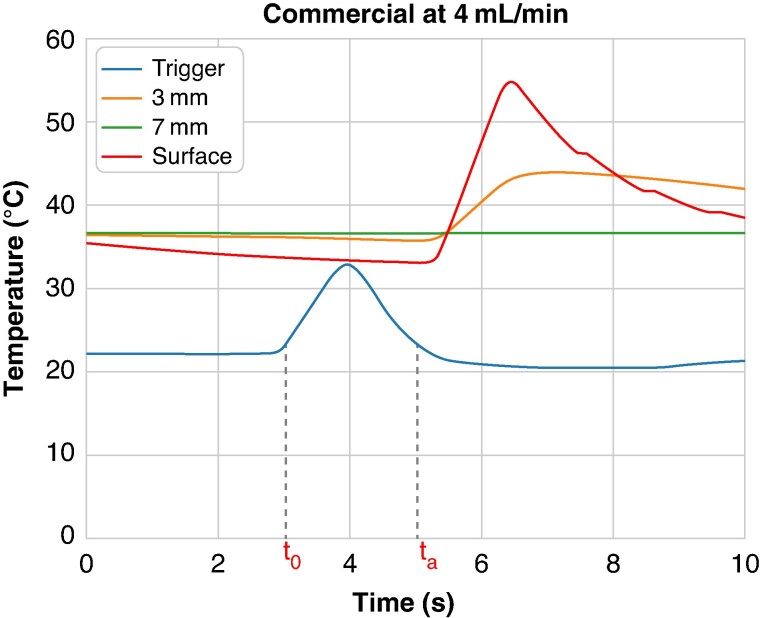
Triggering scheme for estimation of ablation initiation: pedal activation is synchronized with the temperature response of the trigger probe. This activation is defined at the time (*t*_0_) when the trigger signal reaches a value of 10% above baseline. Ablation starts at *t* = *t*_a_, 2 s after pedal activation (*t*_0_).

To estimate the time of initiation of ablation, a fourth fiberoptic-based fluoroptic temperature probe was used. This probe was initially held outside the saline bath, then, manually and rapidly, inserted into the saline bath in synchronization with the activation of the pedal. Temperature probe verification was conducted a day before testing initiation. Both the pedal and probe were operated by the same operator. The time evolution of the probe signal was used as a triggering reference for the ablation. Activation of the pedal was set at time *t* = *t*_0_, defined as the moment when the temperature revealed by the fourth pre increased by 10% above baseline (*Figure 4*). The generator software was preset for ablation initiation with a 2 s delay after the pedal activation for both ablation sequences used. Ablation initiation time (*t*_a_) was calculated 2 s after *t*_0_. The temperature acquisition system used 0.5 mm fiberoptic-based fluoroptic temperature probes and an acquisition system able to sample at 50 Hz (sampling interval: 20 ms).

### Test procedure

For each ablation protocol, both at 4 and 30 mL/min irrigation rates, the median maximum temperature and the corresponding temperature rise (delta) from baseline were reported at three different tissue depths: surface, 3, and 7 mm. The median maximum temperature was calculated for all the peak temperatures observed during each ablation. An ablation was defined as the delivery of a train of 3 pulses (delivered over 20.6 s) for Sequence 1 and 4 pulses (delivered over 3.8 s) for Sequence 2, respectively. A total of 34 ablations were performed for Sequence 1 at 4 mL/min, 32 for Sequence 1 at 30 mL/min, and 33 for Sequence 2 at both 4 and 30 mL/min (*Table 2*).

**Table 2:** Temperature profiles of different ablation settings

Tissue location	Sequence	Irrigation rate (mL/min)	Maximum temperature (°C)	Change in temperature from 37°C (Δ*T*)	Number of ablations
Surface	Sequence 1 (commercial)	4	56.4 (54.9–58.4)	19.4 (17.9–21.4)	34
		30	48.4 (45.2–52.2)	11.4 (8.2–15.2)	32
	Sequence 2 (fast)	4	53.1 (51.7–54.3)	16.1 (14.7–17.3)	33
		30	40.8 (37.9–43.0)	3.8 (0.9–6.0)	33
3 mm	Sequence 1 (commercial)	4	42.5 (41.1–44.3)	5.5 (4.1–7.3)	34
		30	40.0 (39.5–40.9)	3.0 (2.5–3.9)	32
	Sequence 2 (fast)	4	40.8 (39.7–41.7)	3.8 (2.7–4.7)	32
		30	39.6 (39.1–40.3)	2.6 (2.1–3.3)	33
7 mm	Sequence 1 (commercial)	4	37.5 (37.2–37.6)	0.5 (0.2–0.6)	34
		30	37.2 (36.9–37.5)	0.2 (−0.1 to 0.5)	32
	Sequence 2 (fast)	4	36.8 (36.7–37.0)	−0.2 (−0.3−−0.1)	33
		30	36.8 (36.7–36.9)	−0.2 (−0.3−−0.1)	33

### Statistical analysis

Normality of numerical variables was assessed using the Shapiro–Wilk test. All results for non-normally distributed continuous numerical variables were reported as median and interquartile range (IQR). Since most variables significantly deviated from a normal distribution (*P* < 0.05), non-parametric statistical tests were applied. Differences among the four experimental groups, identified by irrigation sequence (Sequence 1 vs. Sequence 2) and flow rate (4 mL/min vs. 30 mL/min), were analysed using the Kruskal–Wallis test. Where significant differences were detected (*P* < 0.05), pairwise Wilcoxon rank-sum tests were performed as post-hoc comparisons. Bonferroni adjustment was applied to all pairwise *P*–values to correct for multiple comparisons. A significance threshold of *α* = 0.05 was adopted throughout. Group differences were visually represented using box plots, which provided a summary of the distribution of temperature and delta values across groups and tissue depths. All statistical analyses were performed using R software version 4.5.0 (Released 11 April 2025).

## Results

### Temperature profile

#### Surface level temperature dynamics

At the tissue surface, the commercial Sequence 1 at a 4 mL/min irrigation rate showed the highest maximum temperature (56.4 [54.9–58.4]) and the largest increase from baseline (19.4 [17.9–21.4]), with observed peak temperatures above 60°C. In contrast, the use of the fast Sequence 2 at 4 mL/min resulted in lower temperatures and reduced increases from baseline when compared to Sequence 1 at the same irrigation rate (53.1 [51.7–54.3]°C vs. 56.4 [54.9–58.4]°C, *P* < 0.001; Δ*T* = 16.1 [14.7–17.3] vs. 19.4 [17.9–21.4], *P* < 0.0001) (*Tables 2* and *3*). When comparing irrigation rates within the same sequence, increasing the flow from 4 to 30 mL/min significantly reduced the temperature:

- At a 30 mL/min irrigation rate setting, maximal temperature and Δ*T* rise observed for the commercial Sequence 1 were statistically lower than those observed at a 4 mL/min setting (48.4 [45.2–52.2], *P* < 0.0001, Δ*T* = 11.4 (8.2–15.2), *P* < 0.0001);
- Also, the fast Sequence 2 at an irrigation rate of 30 mL/min resulted in significantly lower temperatures compared to its use at 4 mL/min (45.9 [44.2–47.9]°C vs. 49.7 [47.9–50.8]°C, *P* < 0.001).

**Table 3:** Wilcoxon rank-sum (Mann–Whitney *U*) test results for thermal parameter comparisons between experimental conditions

Thermal parameter	Comparison	Adjusted *P*-value	Thermal parameter	Comparison	Adjusted *P*-value
Maximum temperature (°C) surface	Seq1_30 vs. Seq1_4	*<0.0001*	Change in temperature from 37°C (Δ*T*) surface	Seq130 vs. Seq1_4	*<0.0001*
	Seq2_4 vs. Seq1_4	*<0.0001*		Seq2_4 vs. Seq1_4	*<0.0001*
	Seq2_30 vs. Seq1_4	*<0.0001*		Seq2_30 vs. Seq1_4	*<0.0001*
	Seq2_4 vs. Seq1_30	*<0.001*		Seq2_4 vs. Seq1_30	*<0.001*
	Seq2_30 vs. Seq1_30	*<0.001*		Seq2_30 vs. Seq1_30	*<0.001*
	Seq2_30 vs. Seq2_4	*<0.0001*		Seq2_30 vs. Seq2_4	*<0.0001*
Maximum temperature (°C) 3 mm depth	Seq1_30 vs. Seq1_4	*<0.0001*	Change in temperature from 37°C (Δ*T*) 3 mm depth	Seq1_30 vs. Seq1_4	*<0.0001*
	Seq2_4 vs. Seq1_4	*<0.01*		Seq2_4 vs. Seq1_4	*<0.01*
	Seq2_30 vs. Seq1_4	*<0.0001*		Seq2_30 vs. Seq1_4	*<0.0001*
	Seq2_4 vs. Seq1_30	*>0.05*		Seq2_4 vs. Seq1_30	*>0.05*
	Seq2_30 vs. Seq1_30	*>0.05*		Seq2_30 vs. Seq1_30	*>0.05*
	Seq2_30 vs. Seq2_4	*<0.01*		Seq2_30 vs. Seq2_4	*<0.01*
Maximum temperature (°C) 7 mm depth	Seq1_30 vs. Seq1_4	*>0.05*	Change in temperature from 37°C (ΔT) 7 mm depth	Seq1_30 vs. Seq1_4	*>0.05*
	Seq2_4 vs. Seq1_4	*<0.0001*		Seq2_4 vs. Seq1_4	*<0.0001*
	Seq2_30 vs. Seq1_4	*<0.0001*		Seq2_30 vs. Seq1_4	*<0.0001*
	Seq2_4 vs. Seq1_30	*<0.05*		Seq2_4 vs. Seq1_30	*<0.05*
	Seq2_30 vs. Seq1_30	*<0.001*		Seq2_30 vs. Seq1_30	*<0.001*
	Seq2_30 vs. Seq2_4	*>0.05*		Seq2_30 vs. Seq2_4	*>0.05*

**Figure 5:**
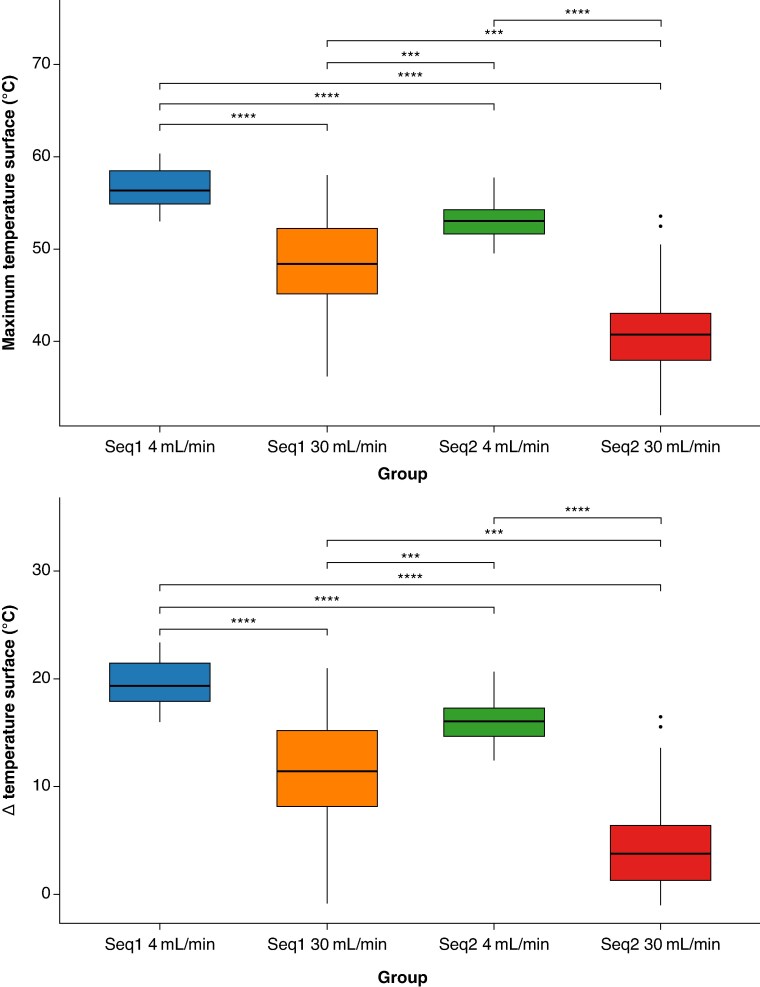
Box plots showing the temperature profile comparison across different ablation settings at the tissue surface level: (top) Maximum surface temperature and (bottom) corresponding temperature increase (Δ*T*, °C) for commercial Sequence 1 and the fast Sequence 2 at 4 mL/min and 30 mL/min irrigation rates. All group comparisons yielded statistically significant differences. Refer to the main text for detailed statistical values and interpretations. N.B.: Box plots display the median, interquartile range, and outliers—black dots. Significance levels: ***P < 0.001; ****P < 0.0001.

Overall, the fast Sequence 2 at a 30 mL/min irrigation rate achieved the best thermal profile across all tested conditions (*P* < 0.001, *Tables 2 and 3* and *Figure 5*).

### 3 mm depth temperature dynamics

**Figure 6:**
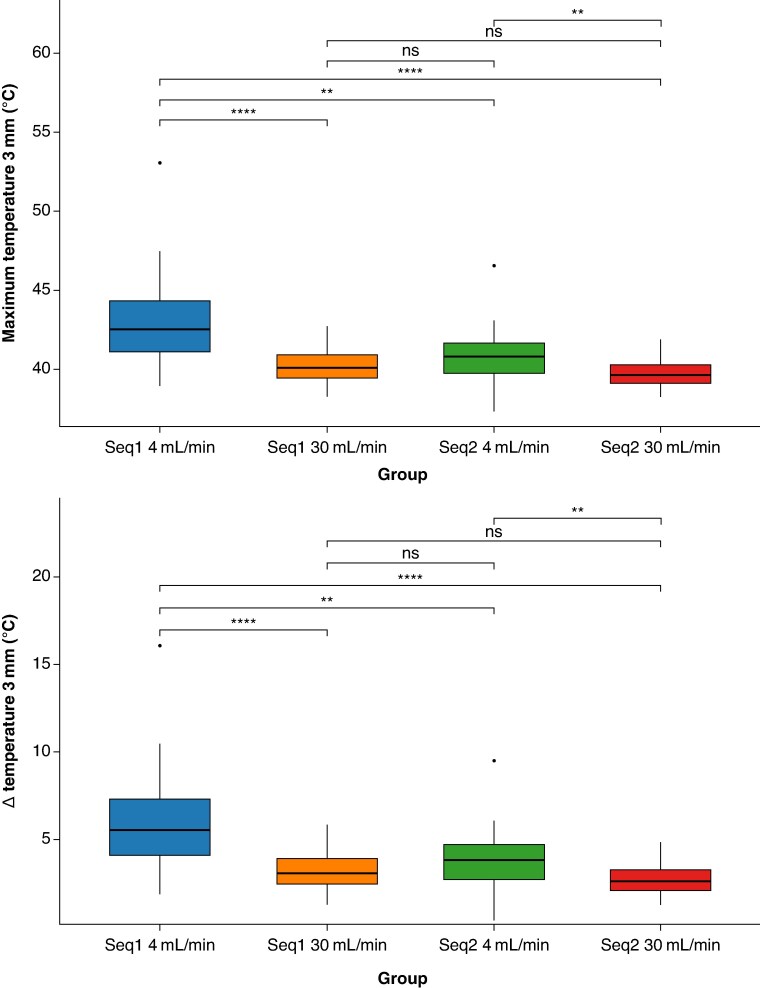
Box plots showing the temperature profile comparison across different ablation settings at 3 mm depth: (top) Maximum temperature and (bottom) relative Δ*T* rise: no significant differences were found between commercial Sequence at 30 mL/min and fast sequences, independently of the irrigation rate used. Commercial Sequence 1 at 4 mL/min showed again the worst thermal outcomes. N.B.: Box plots display the median, interquartile range, and outliers—black dots. Significance levels: **P < 0.01; ****P < 0.0001; ns: non-significant differences.

At 3 mm depth, temperature elevations were generally reduced compared to the surface (*Table 2*, *Figure 6*). The commercial Sequence 1 at 4 mL/min again produced the highest thermal values (42.5 [41.1–44.3]°C, Δ*T* 6.1 ± 2.9 °C). Irrigation increase from 4 to 30 mL/min was effective in both sequences in preventing further thermal increases (*P* < 0.001). The use of the fast Sequence 2 at 30 mL/min was associated with a statistically significant reduction in both maximum temperature and the Δ*T* rise at a depth of 3 mm, compared to the commercial Sequence 1 (38.9 [36.9–40.3]°C vs. 42.5 [41.1–44.3]°C, *P* < 0.001) and the fast Sequence 2 at a 4 mL/min irrigation rate (41.3 [40.1–42.57]°C, *P* < 0.01). The effect was less pronounced when fast Sequence 2 at 30 mL/min was compared to the commercial at the same irrigation rate, with no statistically significant difference observed in either maximum temperature (38.9 [36.9–40.3]°C vs. 40.0 [39.4–40.0]°C, *P* > 0.05) or Δ*T* rise (2.6 [2.1–3.3] vs. 3.0 [2.5–3.9], *P* > 0.05).

### 7 mm Depth temperature dynamics

**Figure 7:**
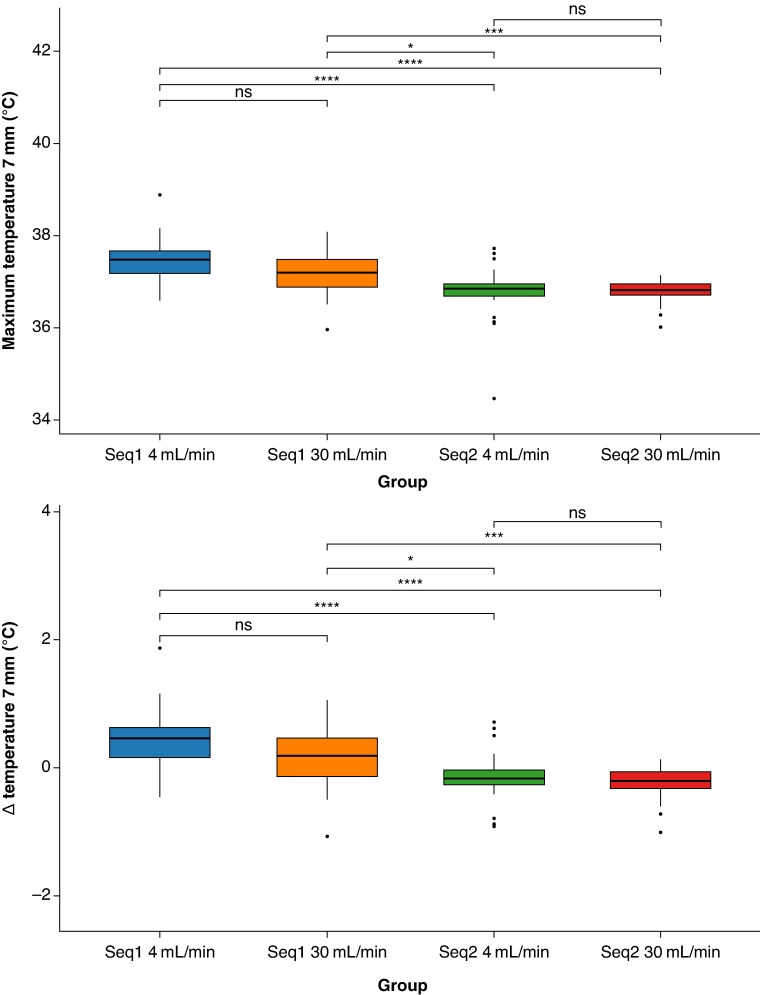
Box plots showing the temperature profile comparison across different ablation settings at 7 mm depth: (top) Maximal temperatures reached were closer to the baseline and (bottom) Δ*T* rise was minimal compared to more superficial layers. Irrigation flow rate did not appear to provide additional cooling benefit at this depth, for either the commercial or the fast sequence. In contrast, the type of ablation sequence used had a stronger influence, with fast Sequence 2 consistently associated with more favourable thermal outcomes. N.B.: Box plots display the median, interquartile range, and outliers—black dots. Significance levels: *P > 0.05; ***P < 0.001; ****P < 0.0001; ns: non-significant differences.

At 7 mm, the thermal rise was minimal across all settings, with tissue temperatures close to baseline (*Table 2*, *Figure 7*). Although differences among the various ablation settings were less pronounced at this depth, the effects of thermal cooling were still evident. Notably, the thermal benefits associated with the ablation sequence appeared to outweigh those conferred by irrigation rate, as reflected in both maximum temperatures and Δ*T* values. Specifically, the fast Sequence 2 at 30 mL/min exhibited the most favourable thermal profile, showing significantly lower temperatures compared to the commercial Sequence 1 at both 4 mL/min (36.4 [36.1–36.7]°C vs. 37.5 [37.2–37.6]°C, *P* < 0.001) and 30 mL/min (37.2 [36.7–37.6]°C, *P* < 0.01). A similar trend was observed for the fast Sequence 2 at 4 mL/min, which demonstrated lower maximum temperatures and smaller Δ*T* increases, compared to the commercial Sequence (*P* < 0.001 and *P*  *<* 0.05, with irrigation rates of 4 and 30 mL/min, respectively). No significant differences were found between the commercial Sequences 1 at 4 and 30 mL/min (*P* > 0.05), or between the fast Sequences 2 at 4 and 30 mL/min (*P* > 0.05).

### Overall comparison by ablation sequence and irrigation rate

**Figure 8:**
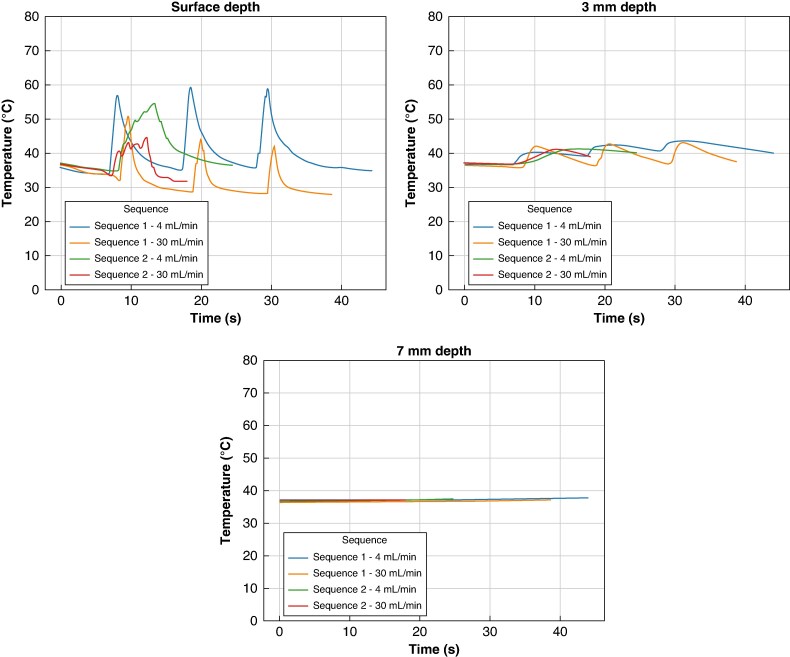
Representative curves of the time evolution of the tissue temperatures at tissue surface, 3, and 7 mm depth during PF ablations with both the tested sequences at 4 mL/min and 30 mL/min.

**Figure 9:**
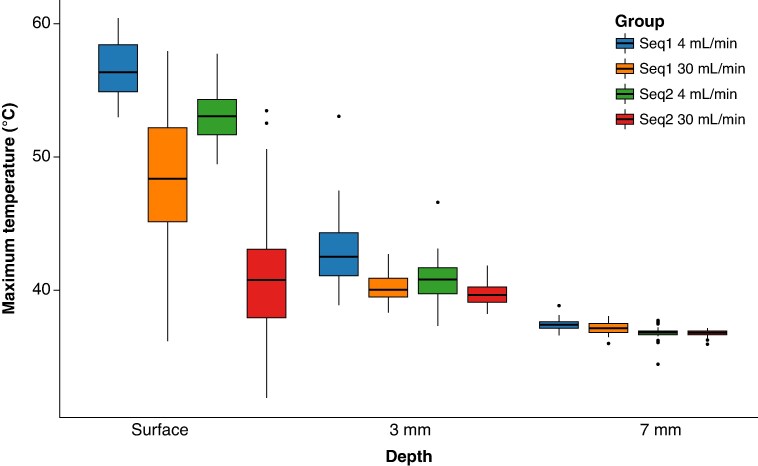
Maximum tissue temperatures at different depths by ablation configuration: box plots display the distribution of maximum tissue temperatures recorded at the surface, 3, and 7 mm depths during PFA using either the commercial 1 or experimental fast 2 pulse waveform at two irrigation flow rates (4 and 30 mL/min). Across all depths, the combination of fast waveform 2 and 30 mL/min irrigation resulted in the lowest maximum temperatures. Notably, surface temperatures were significantly higher than those at 3 and 7 mm, with thermal attenuation observed with increasing depth. Despite reduced absolute values at 7 mm, fast Sequence 2 consistently showed more favourable thermal behaviour compared to the commercial configuration. PFA, pulsed field ablation.

The time evolution of surface temperatures was greatly affected by irrigation, and surface temperature was quickly suppressed immediately after the end of each ablation (*Figure 8*). Temperatures at 3 mm were less affected and followed a smoother time evolution with an incremental increase with increasing number of applications. Temperatures at 7 mm depth showed a minor monotonic increase during ablation. Across all tested conditions, the combination of fast Sequence 2 and 30 mL/min irrigation rate consistently demonstrated the most favourable thermal profile (*Figure 9*). This setting yielded the lowest maximum temperatures and smallest Δ*T* rise values at all tissue depths—surface, 3, and 7 mm—highlighting a synergistic effect between optimized pulse waveform and active cooling.

In contrast, the commercial Sequence 1 at 4 mL/min produced the highest thermal stress, with significantly elevated temperatures and thermal rises, particularly at the surface and at 3 mm depth.

When comparing irrigation rates within the same waveform:

- Increasing irrigation from 4 to 30 mL/min resulted in a clear reduction in both absolute temperature and Δ*T* for both sequences.
- However, waveform optimization (fast vs. commercial) showed a more consistent and greater impact on thermal reduction than irrigation rate alone, especially at deeper layers.

At 7 mm depth, all ablation settings showed minimal temperature elevation, confirming a limited subsurface thermal spread independent of sequence or irrigation. Nonetheless, the fast Sequence 2 maintained a slight thermal advantage over the commercial configuration even at this level.

## Discussion

The results of our study align with previously published literature in confirming that PFA can exhibit a non-negligible collateral thermal profile. In our study, among the two tested configurations, the commercial Sequence 1 consistently resulted in the worst thermal outcomes, with peak superficial temperatures exceeding 60°C at a 4 mL/min irrigation rate. In a previous experimental study, Verma *et al.*^12^ validated the temperature changes at several tissue depths using a different PFA ablation system (Centauri; Galvanize Therapeutics, San Carlos, CA, USA) connected to 3 commercially available focal ablation catheters designed for RF ablation. The authors described increases of 7.6°C at the surface, 2.8°C at 3 mm depth, and 0.9°C at 7 mm depth. These data, in combination with overall absolute temperatures below 50°C, suggested the presence of a mild collateral heating for PFA, when compared to RF ablation. In contrast, in our study, we measured significantly higher peak temperatures and degree of temperature rise from baseline. The main factors we believe may have accounted for the observed differences are represented by the distinct catheter design and the pulse waveform used. Acting synergistically, these two elements confer unique electric field properties to each PFA system, likely resulting in a specific thermal footprint.^3,11^

Moreover, methodological differences in temperature sampling may have contributed to this discrepancy. In the study by Verma *et al.*, the temperature probes were positioned perpendicularly to the tissue at various depths, a configuration that may have placed them some distance away from the actual hotspot. On the contrary, we used a standardized angled insertion to align the sensing probe with the centre of the electrode. This method ensured accurate spatial correspondence with the site of maximal energy and may have offered a more sensitive assessment of the thermal effects associated with PFA delivery. To the best of our knowledge, this is the first study to report such a thermal effect associated for a commercial PFA system routinely employed in daily clinical practice.

There is emerging evidence of an increased rate of neurovascular events associated with PFA, which may be related to thrombus formation due to endothelial injury and/or char development at the electrode–tissue interface. Charring most commonly depends on thermal coagulation and has been described to potentially occur following heat-induced cell necrosis (typically reported for tissue temperature above 50°C).^15^ Once the desiccation point of the tissue has been reached, further energy delivery at the tissue-catheter interface may cause localized over-heating and potentially lead to tissue charring. Traditionally, thrombus formation and charring have been described with irrigated RF catheters at tissue temperatures above 80°C and for catheter–tissue interface temperatures around 100°C.^16^ These values are considerably different from those observed in our study.

Nevertheless, it is important to underline that our measurements were obtained following ablation protocols consisting of only one application (each one based on three to four pulses). In real-world settings, catheter ablation with the VLCC may require up to 16 applications for pulmonary vein isolation (≥4 per vein), with additional lesion sets in the case of more extensive ablation procedures.^17,18^ Furthermore, ablation ‘stacking’—placing multiple overlapping lesions—is a technique often used in clinical practice to enhance lesion efficacy and improve long-term durability. Given the collateral thermal effects associated with each PFA pulse, it is plausible that the catheter might not have fully cooled in the timeframe between consecutive applications. This could potentially lead to a cumulative thermal effect, resulting in a greater-than-expected temperature rise, especially at the electrode–tissue interface. In their recently published study, Sauer *et al.*^19^ addressed this issue by evaluating the thermal implications of consecutive high-energy PFA pulses delivery with the VLCC. The authors demonstrated significant heating of metal electrodes with temperatures reaching up to 86.0°C. While these findings are consistent with our hypothesis, it is important to underline several limitations of this study, which may limit the generalizability of its conclusions. First, temperature assessment has been performed using thermal cameras, a method that can be prone to inaccuracies due to emission assumptions and reflective artifacts.^20^ Besides, the thermal analysis was limited to the surface of the metallic electrodes, without exploring either the electrode-tissue interface or the subsurface tissue temperature profile. Notably, catheter electrodes may differ from the actual tissue temperature, and therefore, the degree of overheating reported by the authors could not be entirely considered a reliable quantitative surrogate for the underlying tissue. Although limited by the experimental setting, these observations suggest a substantial increase in the Joule heating effect and inadequate cooling after successive PFA applications.

Interestingly, when the thermal behaviour was analysed at subsurface layers, moderate temperature rises were also observed at 3 mm depth, while temperatures at 7 mm remained largely unaffected, independently of the ablation sequence or the irrigation velocity adopted. The limited spatial thermal spread observed for PFA might contribute to preventing some of the thermal complications typically associated with heating, such as steam pops and cardiac wall perforation.^21^

Nevertheless, factors such as catheter-to-tissue contact and tissue proximity should also be considered, as they may influence the extent of deeper tissue heating. Mathematical modelling has indicated that PFA-related oesophageal heating is theoretically possible under certain conditions, primarily driven by pulse duration and further modulated by voltage, number and timing of applications, blood–tissue heat exchange, and anatomical proximity.^22^ A variable degree of collateral heating, with modest—mostly clinically insignificant—rises in oesophageal luminal temperature, has also been previously reported.^13^ However, evidence from endoscopic studies and large clinical registries indicates that temperature increases, even when present, do not result in clinical sequelae, thereby reinforcing the overall favourable oesophageal safety profile of PFA.^23–25^ This can be explained by the fact that heat generation depends on the electrical field density, which decreases as a function of distance from the energy source.^26^ Consequently, more distant regions are less likely to experience higher temperature changes. This is consistent with our study observations, where the impact of different energy and irrigation settings on the temperature profile diminishes progressively with increasing tissue depth.

Importantly, the degree of catheter-to-tissue contact may further modulate local thermal behaviour. A greater surface of contact can theoretically reduce heat dissipation into the circulating blood pool, thereby concentrating energy delivery within the tissue and lowering heating at the catheter–blood interface, ultimately reducing the risk of charring. Conversely, suboptimal contact may favour localized overheating at the electrode–blood boundary, where heat is less efficiently dispersed, thus increasing the likelihood of surface charring.

In our experimental setting, we sought to isolate thermal behaviour across four conditions while controlling for tissue contact by applying a standardized 30 g force in every case. Although this approach may limit the evaluation of variable contact-force effects, it reproduces clinically desirable ablation conditions,^27^ and may therefore provide a more reliable representation of the thermal behaviour than that observed with suboptimal or excessive contact. However, the absence of a controlled lesion-stacking model and the use of a bovine ventricular preparation—which lacks the three-dimensional anatomical relationship between the atrium and oesophagus—restricts the extrapolation of these findings to the oesophageal safety. In the absence of direct evidence regarding contact level, application parameters, and thermal effects, these observations should be considered hypothesis-generating rather than conclusive.

In several preclinical studies, it has also been shown that pulse repetition and tissue contact do impact on PFA lesion depth and durability.^28–31^ Based on what is reported on collateral Joule heating during PFA,^6^ it is reasonable to question whether lesion formation can be attributed exclusively to electroporation, or whether thermal contributions also play a role, and to what extent. Recently, Di Biase *et al.*^27^ provided experimental data using a VLCC on bovine myocardium to explore the dose–response relation between the contact force, the number of ablations/ablations, and the characteristics of the lesions achieved. Notably, the authors showed that when using a contact force of 30 g—consistent with the value employed in our experimental setup—it was possible to achieve significantly deeper and broader lesions with the same number of delivered pulses. Conversely, under suboptimal contact conditions (<30 g), a substantially higher number of applications was required to obtain comparable lesion dimensions. Interestingly, despite maintaining a contact force of 30 g, the authors found that lesion depth reached a plateau, with only marginal increases (<1 mm) even when the number of stacked applications was tripled. This plateau effect suggests that the tissue response to repeated applications may reach a saturation point of the electroporative effect, and that ultimately lesion depth is determined by the distance of the targeted tissue from the ablation source. Nakagawa *et al.*^32^ further investigated the role of contact force, showing that values exceeding 30 g were associated with deeper lesions and greater irreversible cellular injury.^32^ Importantly, they reported that in no case thermal damage was observed, even at higher contact forces. This evidence seems to suggest that during PFA, increasing contact may enhance lesion depth by exposing a larger amount of tissue to the electric field at a given density. In contrast, in RF ablation, the enlargement of the lesion with increasing contact force is explained by enhanced heat transfer, with lesion propagation being entirely heat-dependent and strongly influenced by the phenomenon of thermal latency.^33^ In this regard, Nies *et al.*^5^ demonstrated that even when the oesophagus was displaced into direct contact with the PFA catheter, only superficial, non-transmural lesions were observed and completely healed within two weeks. Interestingly, histological analysis consistently revealed mucosal and vascular sparing, reflecting a likely non-thermal, reversible injury limited to the outer oesophageal layers.

This consideration may be particularly relevant in the context of ventricular ablation, where PFA has already proven to be a feasible and acutely effective strategy.^34^ In these demanding settings, electroporation may offer a distinct advantage by enabling adequate lesion formation even in scarred or anatomically complex substrates, where the effectiveness of thermal ablation is limited.^35^ In addition, collateral heat damage could pose a significant safety concern, especially when ablation targets areas in close proximity to critical structures such as coronary arteries or the conduction system. Careful control of pulse delivery and adequate cooling strategies may therefore play an essential role not only in ensuring procedural safety, but also in preserving the efficacy of PFA in the ventricular myocardium.

In our experience, both the modification of pulse parameters and the implementation of active cooling resulted in optimization of the PFA temperature profile. The use of the fast Sequence 2 generated less heat both at the surface level and in deeper layers. Usually, a higher number of pulses within a pulse train has been shown to increase tissue temperature under identical voltage settings,^36^ so one could logically expect that Sequence 2, given the higher number of pulses, should have produced higher overheating when compared to Sequence 1. However, both the duration of the individual pulse and the ‘duty’ cycle used are critical factors influencing the thermal profile.^6,37^ Additionally, the increase in the number of pulses required to produce a substantial thermal difference between the two sequences at constant current and voltage is significantly greater than the modest increase introduced in the new ablation sequence.^36^ Therefore, although the Sequence 2 protocol is characterized by more pulses, it is reasonable to assume that this increase has had a lesser impact on electric field density compared to the beneficial effects of reduced pulse duration and shorter ablation time. These factors likely contributed to lower total energy delivered to the tissue and, consequently, reduced localized heat generation.

When analysing the effect of convective cooling with increased irrigation, we also noticed a reduction in absolute and relative temperature across all the tissue depths, both for Sequence 1 and Sequence 2. The degree of reduction observed was higher when applying the highest irrigation rate to Sequence 2, thus suggesting a synergistic effect of modified waveform and optimized active cooling on Joule heating. Nevertheless, it is noteworthy that adjusting the irrigation settings alone allowed Sequence 1 to achieve a significant temperature decrease, bringing it almost below a threshold of clinical concern. These findings are similar to what has been found by Sauer *et al.*,^19^ who also demonstrated that active cooling with 30 mL/min irrigation rate could help mitigate the thermal rise due to PFA-related resistive heating. Despite this, it must be noted that we observed that commercial Sequence 1 at 30 mL/min consistently demonstrated inferior—and only occasionally comparable—thermal outcomes relative to the fast Sequence 2 at 30 mL/min. Therefore, modification of irrigation rate settings when using the commercial Sequence 1 should be considered a transitional strategy until the new ablation waveform is implemented in routine clinical practice. This protocol refinement would likely permit greater procedural safety, further minimizing collateral heat-related side effects when using VLCC, especially in more extensive ablation procedures. The novelty of our analysis lies in recognizing the critical role of pulse characteristics and the potential to significantly improve the temperature profile of PFA systems through modifications to the waveform. Furthermore, we demonstrated that adjustments to irrigation settings can mitigate not only superficial but also deeper thermal effects. In this sense, our findings offer a complementary and validating perspective to existing studies, providing essential insights that would otherwise remain unaddressed.

### Limitations

One potential limitation of our study is that temperature profile assessment has been performed in a static experimental environment, in the absence of circulating blood or saline solution flow. Notably, a previous study on open-irrigated RF catheters demonstrated that neither high (1 m/s) nor low blood flow (0.5 m/s) significantly influenced the thermal profile at the tissue-catheter interface, suggesting that interface cooling was independent of the local blood flow.^16^ Therefore, it is unlikely that the lack of perfusion in our setup would drastically alter the observed results when translated to the clinical setting, especially considering that the VLCC can be assimilated to an open-irrigated catheter. Additionally, we didn’t systematically explore intermediate flow rate values, but we only provided the two extreme conditions. Future work could include systematic evaluation of intermediate flow rates to confirm whether subtle flow-dependent effects occur, although we believe that these changes are unlikely to significantly alter the tissue temperature in a clinically meaningful way.

Secondly, an additional important consideration concerns the lack of temperature profile validation following consecutive pulse applications and varying contact force. These aspects, which were not fully addressed in our study, may limit the extent to which our temperature measurements capture the true cumulative thermal burden encountered during clinical procedures, potentially leading to underestimation. Finally, it is important to emphasize that the relevance of our findings is confined to the thermal behaviour of the VLCC system. Each PFA platform—depending on its electric field characteristics, which are in turn determined by pulse waveform, catheter geometry, electrode size, and spacing—exhibits a distinct thermal profile. Accordingly, our results should not be regarded as definitive or broadly generalizable, and further comparative studies are needed to determine whether similar effects occur across other PFA systems.

## Conclusion

This study provides the first evidence of significant surface and subsurface thermal effects associated with a commercially available PFA system currently deployed in clinical practice. Our findings demonstrated the critical role of pulse waveform and active cooling in PFA thermal outcomes. While the implementation of the new waveform is expected to further enhance ablation safety, irrigation optimization should be used as a temporary strategy. Future implementation of optimized waveform configurations in combination with active irrigation strategies represents a promising approach to mitigate thermal effects during PFA, enhancing procedural safety without compromising efficacy.

## Data Availability

The data underlying this article will be shared on reasonable request to the corresponding author.
